# H-Ras regulation of TRAIL death receptor mediated apoptosis

**DOI:** 10.18632/oncotarget.2091

**Published:** 2014-06-11

**Authors:** Jun-Jie Chen, William P. Bozza, Xu Di, Yaqin Zhang, William Hallett, Baolin Zhang

**Affiliations:** ^1^ Division of Therapeutic Proteins, Office of Biotechnology Products, Center for Drug Evaluation and Research, Food and Drug Administration, Bethesda, Maryland, United States; ^2^ Department of Medical Research, E-DA Hospital, I-Shou University, Kaohsiung 824, Taiwan, Republic of China

**Keywords:** H-Ras, death receptors, TRAIL, apoptosis, cancer drug resistance

## Abstract

TNF-related apoptosis-inducing ligand (TRAIL) induces apoptosis through the death receptors (DRs) 4 and/or 5 expressed on the cell surface. Multiple clinical trials are underway to evaluate the antitumor activity of recombinant human TRAIL and agonistic antibodies to DR4 or DR5. However, their therapeutic potential is limited by the high frequency of cancer resistance. Here we provide evidence demonstrating the role of H-Ras in TRAIL receptor mediated apoptosis. By analyzing the genome wide mRNA expression data of the NCI60 cancer cell lines, we found that H-Ras expression was consistently upregulated in TRAIL-resistant cell lines. By contrast, no correlation was found between TRAIL sensitivity and K-Ras expression levels or their mutational profiles. Notably, H-Ras upregulation associated with a surface deficiency of TRAIL death receptors. Selective inhibition of H-Ras activity in TRAIL-resistant cells restored the surface expression of both DR4 and DR5 without changing their total protein levels. The resulting cells became highly susceptible to both TRAIL and agonistic DR5 antibody, whereas K-Ras inhibition had little or no effect on TRAIL-induced apoptosis, indicating H-Ras plays a distinct role in the regulation of TRAIL death receptors. Further studies are warranted to determine the therapeutic potential of H-Ras-specific inhibitors in combination with TRAIL receptor agonists.

## INTRODUCTION

The outgrowth of cancer cells is a result of deregulated tissue homeostasis, which is tightly regulated through the delicate balance of cell growth and apoptosis [1]. Therefore, a key strategy in the development of anticancer drugs has been to block the aberrant growth signaling components or to directly activate apoptosis machinery in cancer cells [2]. This approach has resulted in several novel cancer therapies including humanized monoclonal antibodies directed against VEGF receptors [3, 4] or ErbB family of receptors [5]. However, these targeted therapies are ineffective for tumors harboring Ras mutations that are found in ~30% of human tumors [6, 7].

In searching for a remedy, a promising strategy has been to target the death receptors (DRs) such as TNFR1 (TNF receptor 1), Fas, DR4 and DR5. These receptors are characterized by an intracellular death domain that transmits a death signal from their respective cognate ligands. TNF-related apoptosis inducing ligand (TRAIL) induces apoptosis through DR4 and/or DR5, and this cytotoxicity appears to be restricted to cancer cells as most normal cell types remain unaffected. The selectivity of TRAIL in killing cancer cells promoted multiple clinical trials to evaluate the potential antitumor activity of recombinant human TRAIL (*e.g.* dulanermin) and agonistic antibodies to DR4 (*e.g.* mapatumumab) or DR5 (e.g. lexatumumab, AMG 655, PRO95780, LBY135, and CS-1008) [2, 8, 9]. These products have a well-tolerated safety profile in the completed Phase I studies [10-13]. However, their therapeutic potential is limited because approximately half of cancer cell lines are resistant to TRAIL receptor-mediated apoptosis [8, 14-18]. An in-depth analysis of resistance mechanisms could facilitate the identification of biomarkers for prediction of tumor response to the DR-targeted therapies and aid in the development of combinational therapies to overcome resistance towards a better clinical outcome of cancer treatment.

The apoptosis signaling through TRAIL death receptors involves several checkpoints [2, 8, 9]. As a prerequisite for ligand binding, the receptors must be expressed on surface membrane wherein it recruits the adapter protein FADD and caspase 8 or 10 into a death inducing signaling complex (DISC). Subsequent activation of downstream caspases leads to cleavage of structural proteins and irreversible cell damage. The caspase activity is subject to regulation by intracellular proteins such as c-FLIP, IAPs and Bcl2 family members. The fate of a cell is also dependent on the status of proliferative proteins (*e.g.* oncogenic Ras).

TRAIL resistance has been linked to genetic or epigenetic alterations in the relevant molecules. These alterations include defects in the TRAIL receptors themselves, e.g. epigenetic silencing of DR4 [19], O- and N-linked glycosylation status [20], and co-existence of decoy receptors [21]. We and others have shown that DR4 and DR5 are absent on surface membrane despite their total protein expressions in various cancer cells [14, 15, 18, 22]. To add complexicity, treatment of cells with repeated doses of TRAIL or anti-DR5 antibody induces a rapid internalization of DR4 and/or DR5 which in turn renders acquired resistance [16-18]. The loss of surface receptors appears to be a major determinant of mechanism of cancer resistance to the DR-targeted therapies. Several intracellular anti-apoptotic proteins (*e.g.* c-FLIP, c-IAPs and Bcl-2 family members) are also found to be elevated in some cancer cell lines wherein they interfere with the caspase signaling cascade (*see* reviews [2, 8, 9]). However, these molecular changes were not broadly applicable to different cancer types.

In this study, we sought to determine if other mechanisms are involved in the development of cancer resistance to the DR4/DR5 agonists. We employed the NCI60 panel of human cancer cell lines representing nine different cancer types. Measured TRAIL sensitivity data were correlated with genome wide mRNA expression data of each of the cell lines. H-Ras was the only gene whose expression levels are significantly higher in TRAIL-resistant cells compared to TRAIL-sensitive cells. Knockdown of H-Ras in TRAIL-resistant cells increases the surface expression of DR4/DR5 and renders the cells susceptible to TRAIL receptor agonists. We conclude that H-Ras is a critical regulator of the dynamics of TRAIL death receptors.

## RESULTS

### H-Ras is upregulated in TRAIL-resistant cancer cell lines

**Figure 1:**
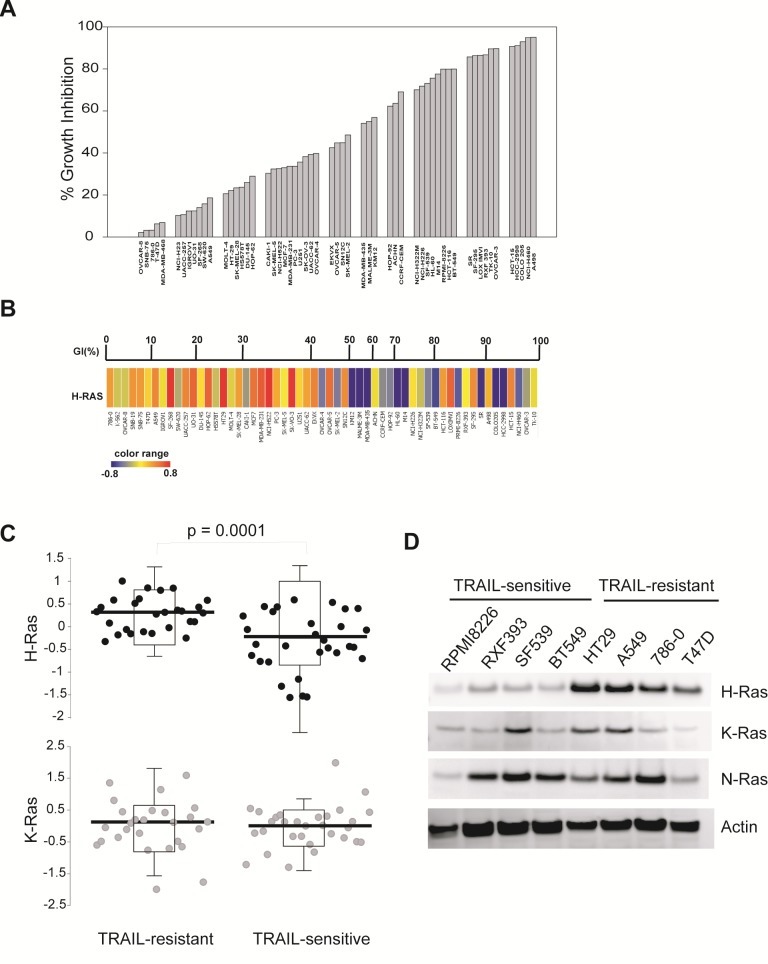
H-Ras is upregulated in TRAIL-resistant cells *A*, Cells were seeded onto 96-well plates and were treated with rhTRAIL at 100 ng/mL for 24 h at 37 °C. Cell viability was measured by SRB calorimetric assay. The percent growth inhibition (GI %) was determined relative to respective untreated cell lines. *B*, H-Ras mRNA expression levels were compared with TRAIL-sensitivity in the NCI60 panel. *Upper panel*, GI% from Fig. 1A; *Lower panel*, blue to red color change indicates a gradual increase in H-Ras mRNA expression level. *C*, The NCI60 panel was divided into two groups, TRAIL-sensitive and TRAIL-resistant, using a cut-off of 50% growth inhibition shown in *A.* The mean expression values of H-Ras or K-Ras between the two groups were tested for significance by a two-tailed Student's t-test. The levels of H-Ras mRNA are significantly higher in TRAIL-resistant lines compared to TRAIL-sensitive lines (p-value = 0.0001). *D*, Western blots of Ras protein expressions in eight randomly selected representatives from the NCI60 panel. Cells were cultured to 70-80% confluency and whole cell lysates were analysed by immunoblotting using antibodies specific to the indicated proteins. Actin was used as a loading control. Shown are representatives of two independent experiments.

We first determined TRAIL-induced cytotoxicity in the NCI60 panel of human cancer cell lines. The NCI60 panel contains 60 human cancer cell lines, representing nine different cancer tissues from leukemia, melanoma and cancers of the lung (non-small cell lung cancer, NSCLC), colon, brain, ovary, breast, prostate, and kidney [24]. As shown in Fig. 1A, these cell lines displayed very different response rates when treated with 100 ng/mL TRAIL for 24 h. Ten cell lines (e.g. A498, NCI-H460) appeared to be highly sensitive to TRAIL induced cell death (>90% growth inhibition). By contrast, twelve cell lines (e.g. OCCAR-8, SNB-75, and A549) were either resistant or slightly responsive to TRAIL (<20%). The majority (38 cell lines) of the panel displayed a modest growth inhibition ~20-80%. No pattern was noted between tumor types and TRAIL sensitivity.

The genome wide mRNA expression data for 58 of 60 of the NCI60 panel were determined by Shankavaram and colleagues [23] and are available at http://www.ncbi.nlm.nih.gov/geo/query/acc.cgi?acc=GSE5720. Taking advantage of this, we searched for genes that are potentially associated with the measured TRAIL sensitivity. By ANOVA analysis (*see* Materials and Methods), we identified H-Ras to be the only gene whose mRNA expression was significantly upregulated in TRAIL-resistant cell lines when compared to TRAIL-sensitive lines (Fig. 1A & B). Previous studies have suggested a role for K-Ras in regulating TRAIL-induced apoptosis [25-29]. However, we found no correlation between K-Ras mRNA levels and TRAIL sensitivity in the NCI60 panel (Fig. 1C).

Next, we determined the protein expressions of H-Ras and its closely related isoforms. To this end, eight cell lines were chosen from the NCI60 based on their different sensitivities to TRAIL treatment (Fig. 1A). Consistent with the mRNA data, the protein expression levels of H-Ras, but not K-Ras or N-Ras, were consistently higher in TRAIL-resistant cell lines when compared to TRAIL-sensitive cell lines (Fig. 1D).

We further evaluated the mutational profiles of Ras genes in each of the sixty cell lines using the published gene expression data [30, 31]. As summarized in Table 1, a dominant-active K-Ras mutation (G12 or Q61) was present in multiple cancer cell lines of the NCI60 panel, irrespective of TRAIL sensitivity. By contrast, H-Ras mutation was only detected in the HS578T breast cancer cell line. Therefore no correlation between K-Ras or H-Ras mutation profiles and measured TRAIL sensitivity was observed. Together, these data demonstrate that the upregulated expression of H-Ras, rather than its oncogenic mutations, is closely linked to TRAIL resistance in a wide range of cancer types.

**Table 1:** Mutation profiles of H-Ras, K-Ras and BARF^1^)

Cell lines^2^)	GI%^3^)	H-RAS	K-RAS	BRAF	Cell lines^2^)	GI %	H-RAS	K-RAS	BRAF
OVARY-8	2				EKVX	42			
786-0	3				SN12C	45			
SNB-19	−1				SK-MEL-2	49			
SNB-75	3				OVARY-5	45		G12V	
T47D	6				MALME-3M	55			V600E
K562	−4				MDA-MB-435	54			
IGROV1	12				KM12	57			
NCI-H23	10		G12C^4^)		CCRF-CEM	69		G12D	
SW-620	16		G12V		ACHN	64			
A549-ATCC	19		G12S		HOP-92	62			
UACC257	11			V600E	NCI-H322M	70			
UO31	12				HL-60	76			
SF-268	14				SF-539	73			
DU-145	26				NCI-H226	72			
SK-MEL28	23			V600E	M14	78			V600E
HT29	22				SR	86			
HOP-62	29		G12C		HCT116	80		G13D	
MOLT-4	21				RXF393	87			
HS578T	24	G12D^4^)			RMPI-8226	80		G12A	
SK-VO-3	38				SF-295	86			
U251	36				LOX IMVI	86			V600E
NCI-H522	32				BT549	80			
MDA-MB-231	36		G13D	G464V^6^)	HCC-2998	91			
MCF7	33				HCT15	91		G13D	
PC3	34				COLO205	93			
CAKI-1	30				NCI-H460	95		Q61H	
UACC-62	39				TK10	90			
SK-MEL5	32			V600E	A498	95			
OVARY-4	40				OVACAR-3	90			

1) Mutation data obtained from [30, 31]

2) NCI60 panel of human cancer cell lines [24]

3) Growth inhibition (GI%) as shown in Fig. 1A.

4) Point mutation at residue 12 or 61 renders Ras GTPases to be constitutively active [54].

### Suppression of H-Ras activity sensitizes TRAIL-resistant cells to TRAIL receptor agonist induced apoptosis

**Figure 2:**
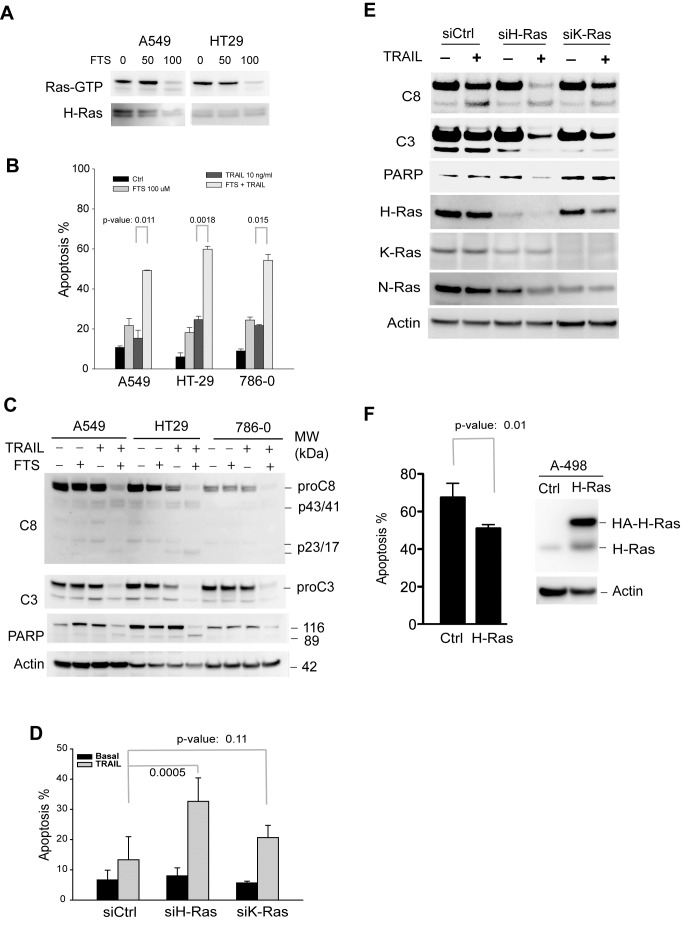
Inhibition of H-Ras activity sensitizes TRAIL-resistant cells to TRAIL-induced apoptosis TRAIL-resistant cell lines, A549, A786-0, and HT29, were randomly chosen for these experiments. *A*, Cells were left untreated or treated at 37°C with (FTS), a pharmacological inhibitor of Ras GTPases, at 50 or 100 μM. After 48 h, cells were harvested and lysed in a buffer. Equal amounts of whole cell lysates were incubated with an antibody specific to the GTP-bound, active form of H-Ras (Ras-GTP). The immunocomplexes were precipitated by protein A agarose beads and blotted using an anti-H-Ras antibody. Equal inputs were confirmed by blotting H-Ras in the whole cell lysates that were used. Ras-GTP was effectively suppressed with 100 μM FTS in 48 h, and therefore this condition was used for all experiments. *B & C,* FTS increases TRAIL induced apoptosis. The indicated cells were left untreated or treated with FTS at 100 μM for 48 h, and then incubated with 10 ng/mL rhTRAIL for an additional 24 h. The resultant cells were analysed by flow cytometry (*B*) for apoptosis or by western blotting for cleavage of caspase-3 and caspase-8 (*C). D & E,* specific isoform knockdown of H-Ras increases TRAIL-induced apoptosis. A549 cells were transiently transfected with a scramble siRNA (siCtrl) or siRNA specific to H-Ras or K-Ras transcript. After 24 h post-transfection, cells were treated with 10 ng/mL rhTRAIL for an additional 24 h. The resultant cells were analysed for apoptosis (D) and caspase cleavage (E). F, A498 cells were transiently transfected with a plasmid encoding wild-type H-Ras. At 24 h post-transfection, cells were treated with 10 ng/mL of TRAIL for additional 6 h and measured for apoptosis by flow cytometry analysis. Shown are representatives of three experiments. The p-values (Student's t test) indicate a significant difference between the two indicated groups.

To define the role of H-Ras in TRAIL-induced apoptosis, we tested the effect of inhibition of Ras activity on TRAIL-induced apoptosis. Three TRAIL-resistant cell lines (A549, HT29, and 786-0) were randomly chosen from the NCI60 panel and were treated with farnesythiosalicylic acid (FTS). It has been shown that FTS inhibits Ras activity by dislodging Ras from the cell membrane thereby rendering it susceptible to proteolytic degradation [32-35]. As shown in Fig. 2A,-Ras activity was effectively suppressed in cells treated with 100 μM FTS for 48 h. This condition was adopted for all experiments. While remaining resistant to TRAIL alone, the cell lines became susceptible to the combination treatment with FTS and TRAIL (Fig. 2B). All three cell lines displayed ~50% of apoptosis under the specified conditions. Consistently, caspase-3 and caspase-8 were activated in the FTS-pretreated cells as indicated by the cleavage of pro-enzymes and the caspase substrate PARP (Fig. 2C). In addition to H-Ras, other Ras family members (e.g. K-Ras and N-Ras) were also shown to be inhibited by the general inhibitor FTS [32, 33]. To determine if K-Ras inhibition was involved in TRAIL sensitization, we blocked individual Ras isoforms by RNA interference approach (Fig. 2D & E). Cells were transfected with siRNA duplexes specific to H-Ras or K-Ras transcript. As a control, duplicate cell samples were transfected with a scramble siRNA. The protein expression of H-Ras and K-Ras was selectively diminished by respective siRNA transfection (Fig. 2E). The siRNA-transfected cells were then treated with TRAIL and analyzed for apoptosis and caspase activation. Knockdown of H-Ras significantly increased TRAIL-induced apoptosis, whereas only a marginal effect was observed when K-Ras was interrupted (Fig. 2D & E). Further, we determined the effect of overexpressing H-Ras on TRAIL induced apoptosis. To this end, A498 cells were transfected with a plasmid encoding wild-type H-Ras. This cell line was chosen because it expresses low levels of endogenous H-Ras and was shown to be hypersensitive to TRAIL (Fig. 1). Notably, overexpression of H-Ras successfully inhibited TRAIL induced apoptosis in A498 cells (Fig. 2F). These data together suggest that H-Ras is selectively involved in the regulation of TRAIL death receptor mediated apoptosis.

**Figure 3:**
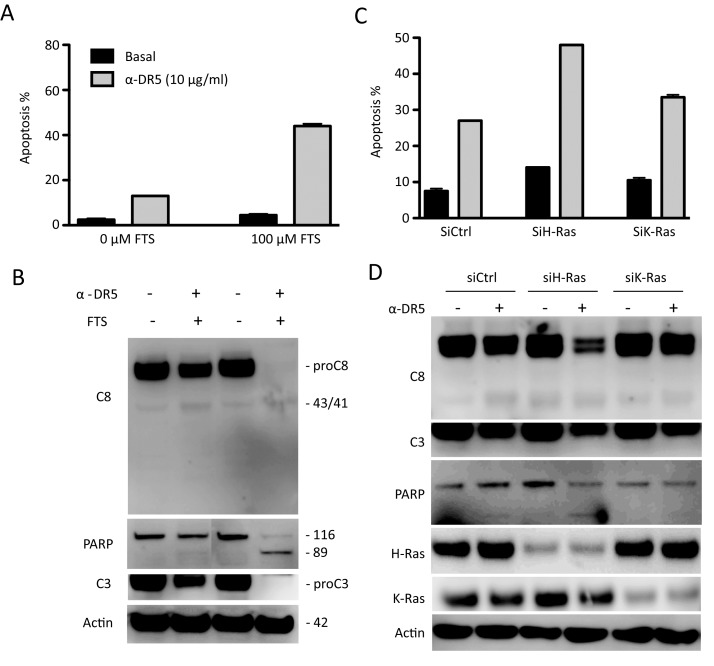
Inhibition of H-Ras renders TRAIL-resistant cells susceptible to anti-DR5 antibody A & B, A549 cells were treated with FTS (100 μM, 48 h) followed by anti-DR5 (10 μg/mL, 24 h). *C & D*, siRNA knockdown (48 h) was followed by anti-DR5 treatment (10 μg/mL, 24 h). Shown are representatives of three experiments.

We asked if Ras inhibition could affect cellular sensitivity to DR5 agnostic antibodies that are being evaluated in multiple clinical trials. Based on the data in Fig. 2, TRAIL-resistant A549 cells were treated with a monoclonal anti-DR5 antibody in the absence or presence of Ras inhibitors. As shown in Fig. 3A & 3B, pretreatment with FTS markedly increased apoptosis by anti-DR5. A similar synergy effect was achieved by silencing H-Ras but not K-Ras transcript (Fig. 3C & 3D). These data together suggest that H-Ras is a critical determinant of cancer resistance to TRAIL receptor targeted therapies. The data also suggest that the associated cancer resistance could be overcome by combining with drugs that function through blocking Ras signaling activity.

### H-Ras downregulates surface expression of TRAIL death receptors

**Figure 4:**
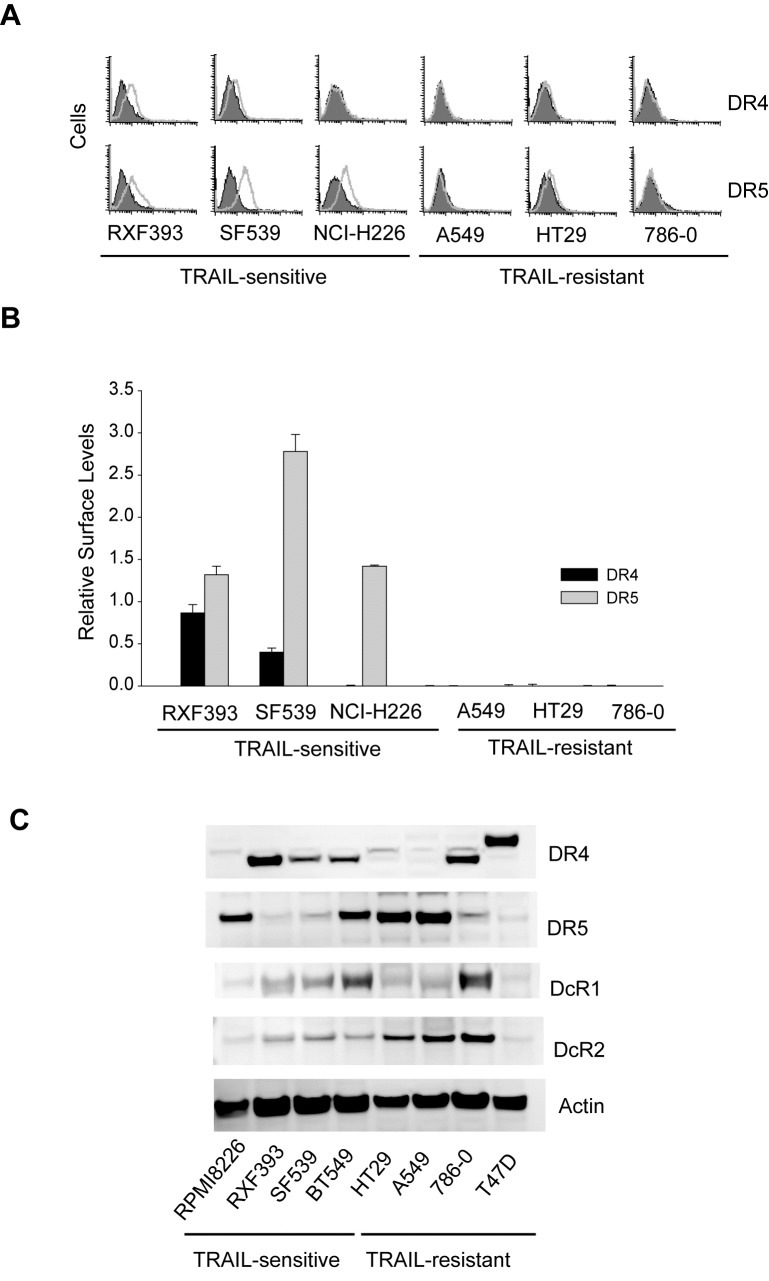
Surface expression of TRAIL death receptors *A*, Surface expression of DR4 and DR5 were determined by flow cytometry using PE-conjugated monoclonal antibody (*open histograms*) or with an isotype-matched control IgG (shadowed histograms). The right-shift indicates the presence of a receptor on cell surface. Shown are representatives of six independent experiments with similar results. *B*, Relative levels of cell surface DR4 and DR5 were estimated by the difference between the mean values of PE-antiDR4 or PE-antiDR5 antibodies and their corresponding IgG-PE controls, divided by that of an IgG-PE control. *C*, Western blots for total protein expressions of TRAIL receptors.

We have previously shown that TRAIL death receptors are absent on the surface membrane of some breast cancer cell lines [14, 18, 36], despite their relatively high total protein expressions. This surface deficiency of DR4/DR5 is sufficient in rendering the cells resistant to the DR-targeted therapies. We asked if this was true for other types of cancer cells on the NCI60 panel. To this end, we performed flow cytometric analysis to monitor surface expression of DR4 and DR5 for six representative cell lines (Fig. 4A & B). Notably, DR4 and DR5 were barely detected on the surface of TRAIL-resistant cell lines (A549, HT29, and 786-0), despite considerable protein expression levels (Fig. 4C). In contrast, DR4 and DR5 were detected on the surface of TRAIL-sensitive cell lines (RXF393, SF539, and NCI-H226) at much higher levels (Fig. 4A & B). These data are in line with our previous observations in breast cancer cells, showing a surface deficiency of TRAIL death receptors in multiple cancer types.

**Figure 5:**
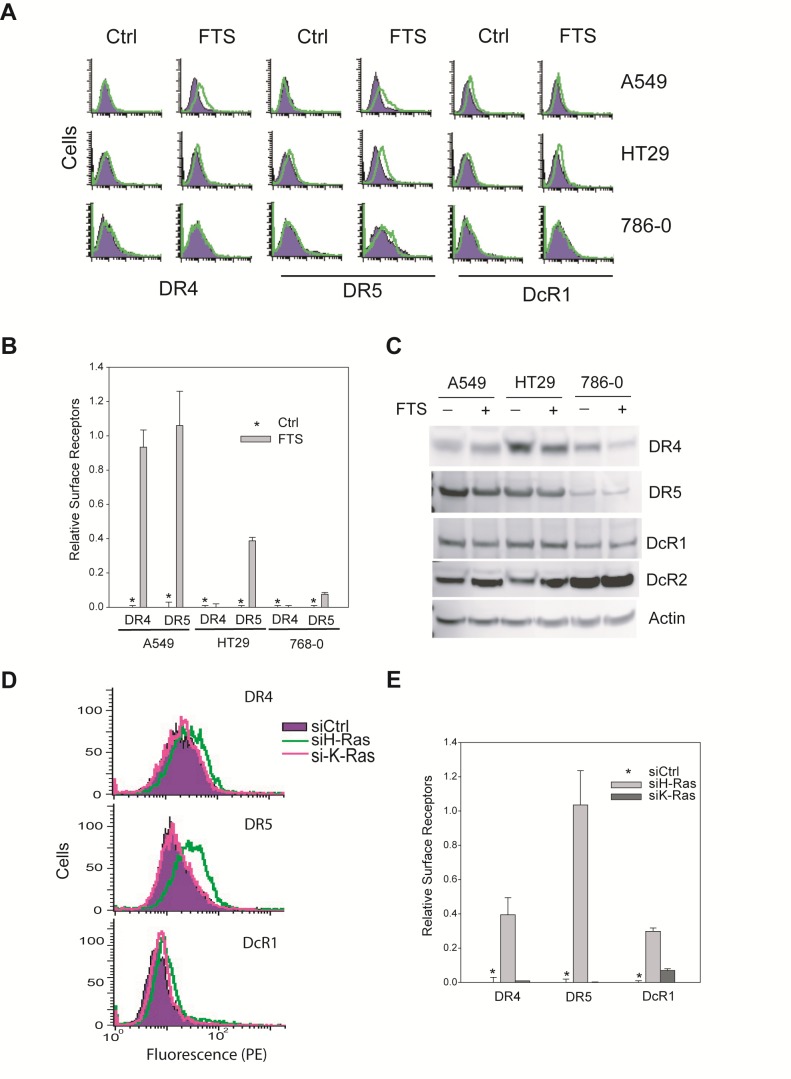
H-Ras inhibition upregulates surface expression of DR4 and DR5 *A,* Cells were treated with FTS at 100 M for 48 h and analysed by flow cytometry using antibodies specific to DR4, DR5 or DcR1. Shown are representatives of histograms comparing surface expressions of TRAIL receptors before and after treatment with Ras inhibitor. *B*, quantification of results in A reveal the relative levels of surface DR4 and DR5. *C*, Western blots showing the total protein expression levels of TRAIL receptors. DR4 was slightly decreased in response to FTS treatment in HT29 and 786-0 cell lines. By contrast, no apparent change was detected for DR5 or decoy receptors. *D*, Knockdown of H-Ras increases surface expression of DR4 and DR5. Cells were transiently transfected with a negative control siRNA (siCtrl) or siRNA against H-Ras or K-Ras. The resultant cells were analysed by flow cytometry for surface DR4 and DR5 as in Fig. 4. Shown are representatives of triplicate experiments. *E*, Relative levels of surface receptors were determined as in 4B (mean ± SD).

So far, we have shown that H-Ras is generally upregulated in TRAIL-resistant cancer cell lines that are also characterized by a surface deficiency of DR4 and/or DR5. This prompted us to test if there was a link between H-Ras and the subcellular localization of TRAIL receptors. Notably, pharmacological inhibition of H-Ras increased the surface expression of DR4/DR5 with a more profound effect on DR5 in the cells examined (A549, HT29, and 768-0) (Fig. 5A & B), while their total protein levels remained essentially unchanged (Fig. 5C). This data suggests that DR4 and/or DR5 were translocated from cytoplasm or intracellular compartments to plasma membrane upon Ras inhibition. As a control, the decoy receptor 1 (DcR1) was not affected in its total protein nor surface abundance. We confirmed this result by specifically targeting H- and K-Ras with siRNA duplexes (Fig. 5D & E). The siRNA transfections were performed as in Fig. 2D and Ras proteins were effectively knocked down by respective siRNA. Upon H-Ras knockdown, the two death receptors, DR5 in particular, were significantly increased on the surface of A549 cells whereas DcR1 was not affected. These data demonstrate that blockade of H-Ras, but not K-Ras, restored the surface expression of TRAIL death receptors. As the total proteins were not altered (Fig. 2E & Fig 5C), the enhanced surface expression of DR4/DR5 is likely a result of redistribution of the receptors from intracellular compartments to the plasma membrane.

We also determined the expressions of proteins that are known to be involved in the regulation of death receptor-mediated apoptosis signaling pathway. These proteins include antiapoptotic proteins (c-IAP1, FLIP, Bcl-2, Bcl-XL, and Mcl-1) and proapoptotic proteins (BAX and BID) (Supplement I). These apoptotic signaling proteins are differentially expressed across the eight representative cell lines, and none of them showed a consistent correlation with the measured TRAIL sensitivity. Collectively, these data provide evidence that H-Ras inhibits TRAIL-induced apoptosis through downregulation of surface DR4/DR5.

## DISCUSSION

The clinical efficacy of cancer therapies is limited by the intrinsic or acquired tumor resistance. Because of this, there are concerted efforts to improve the antitumor activity of DR4/DR5 targeted therapies by rationally designing combinational drugs that would overcome or bypass the resistance mechanisms within the cancer cells. In this study, we show that H-Ras is a key mediator of cancer resistance to TRAIL and anti-DR5 antibody. In the NCI60 panel, H-Ras mRNA levels inversely correlate with surface DR4/DR5 and cellular susceptibility to TRAIL-induced apoptosis. The H-Ras-mediated resistance to TRAIL signaling appears to be a direct result of the loss of DR4/DR5 surface expression, since knockdown of H-Ras effectively restored both surface expression and susceptibility. Our data warrant further studies to evaluate the therapeutic potential of DR-targeted agents in combination with Ras inhibitors or growth factor receptor agonists. Also the upregulation of H-Ras in TRAIL resistant cells provides a potential biomarker algorithm for the prediction of tumor resistance to the DR-targeted therapies. Additionally, our data suggests that H-Ras not only promotes cell proliferation by its oncogenic nature, but also protects cancer cells from cytokines (e.g. TRAIL) mediated surveillance.

H-Ras, together with K-Ras and N-Ras, are the founding members of the Ras family of small GTPases, which are widely implicated in promoting cell proliferation, survival, and transformation [37, 38]. These proteins are membrane-localized GTPases that are activated in response to extracellular signals (e.g. epidermal growth factors) that induce GDP for GTP exchange. In the GTP-bound state, Ras proteins can interact with and activate many downstream effectors including Raf, PI3K and RalGDS [37, 38]. Our data demonstrate that H-Ras is a crucial regulator of death receptor mediated apoptosis pathways. The upregulation of H-Ras correlates with a deficiency of surface DR4 and/or DR5 in TRAIL-resistant cell lines. Notably, blockade of H-Ras activity successfully restored surface expression of both DR4 and DR5 without changing their total protein expression levels. Thus, H-Ras appears to inhibit TRAIL-induced apoptosis by suppressing the surface expression of DR4 and/or DR5.

During our analysis we were also interested in determining if there was any correlation between sensitivity to TRAIL induced apoptosis and mutant K-Ras and H-Ras expression. In the literature to date, the effect of Ras mutation in TRAIL sensitivity has been contradictory. Consistent with our data, recent studies have shown that TRAIL-resistant cells were sensitized by combinatorial treatment of TRAIL with cetuximab (an anti-EGFR monoclonal antibody) or sorafenib (a multi-kinase inhibitor that acts on Raf kinases, MEK, and ERK signaling) in different cancer cell lines and tumor xenograft models [39, 40]. By contrast, several other studies suggested an opposite role for oncogenic Ras in TRAIL-induced apoptosis [25-27, 29, 41, 42]. For example, Drosopoulos et al. [25] showed that transfection of H-Ras12V or K-Ras12V increased TRAIL-induced apoptosis in a colon cancer cell line caco-2. Oh et al [27] showed an increase in DR5 expression and TRAIL sensitivity in HEK293T cells by forced expression of oncogenic Ras (e.g. H-Ras12V or K-Ras12V) or B-RafV600E mutant. In our analysis, approximately 30% of the cell lines in the NCI60 panel express an active K-Ras (residues 12 or 13) and/or B-RafV600E mutant, which are expected to be resistant to anti-EGFR therapies (Table 1). Although some of these cell lines were found to be susceptible to TRAIL-induced apoptosis, there is no clear correlation between TRAIL sensitivity and the status of K-Ras or H-Ras mutations in the NCI60 panel of human cancer cell lines (Table 1). The reasons for these discrepancies are not clear but may be related to the differences in cancer types or experimental approaches used in various labs. All previous studies examined the role of Ras in TRAIL-induced apoptosis by transfection with a constitutively active Ras mutant (e.g. H-Ras12V or K-Ras12V). However, it is known that *in vitro* transfection of H-Ras12V or K-Ras12V can induce cellular stress in cultured cell lines which in turn can promote cell death via senescence or autophagy-mediated pathways [43-45]. This raises a concern about the physiological relevance of data derived from forced expression of Ras mutants, especially in the context of studying TRAIL induced cell death phenotypes. In contrast to these previous studies, we identified H-Ras by correlating TRAIL sensitivity with genome wide mRNA expressions and confirmed the result by specifically silencing H-Ras or K-Ras transcript. It will be interesting to determine how cancer cells discriminate Ras isoforms in response to death receptor activation. Nonetheless, H-Ras upregulation correlates with cancer resistance to TRAIL or anti-DR5 antibody. Our data provide a rational to evaluate the therapeutic potential of combinational therapies that simultaneously target the defects in both EGFR/Ras and TRAIL apoptosis pathways.

Agreeing with our current observation, H-Ras has been shown to inhibit Fas ligand-mediated apoptosis by downregulating the surface expression of Fas [46]. Thus, H-Ras may be broadly involved in regulating the dynamics of death receptors. This distinct function of H-Ras is intriguing given that K-Ras and other isoforms are nearly identical with respect to their catalytic and effector-binding properties [38]. One possibility is related to isoform-specific post-translational modifications [47]. H-Ras is subject to farnesylation of the CAAX site and palmitoylation on two cysteine residues within the C-terminal domain, with both necessary for its stable association with the membranes. K-Ras undergoes only a single lipid modification – farnesylation-and its membrane binding is stabilized by a C-terminal poly-lysine stretch. Additionally, palmitoylation of H-Ras targets it to the Golgi compartment during its biosynthetic route from the ER to the plasma membrane. This modification is also responsible for partitioning of H-Ras to the lipid rafts, a microdomain of the plasma membrane enriched with cholesterol, at the plasma membrane. K-Ras generally does not localize to the Golgi, and it is absent from the lipid rafts and present in the bulk membrane. Interestingly, pharmacological inhibition of palmitoylation (essentially inhibiting H-Ras but not K-Ras) enhanced TRAIL-induced apoptosis in A549 and HT29 cell lines (Supplement II). It is also noted that the frequency of oncogenic mutation in H-Ras is much lower than that of K-Ras in various tumors [48]. Emerging evidence shows that TRAIL sensitivity is related to the distribution of DR4/DR5 between lipid rafts and bulk plasma membrane [40, 49-51]. These data suggest that H-Ras may regulate DR4/DR5 dynamics through post-translational processes such as lipid raft-dependent events. Studies are underway in our laboratory to determine the biochemical pathways that control the temporal and spatial expression of death receptors.

Death ligands (e.g. TRAIL, FasL, and TNF) are cytokines primarily produced by T cells and natural killer cells [52, 53]. These cytokines are present in circulation and tissue microenvironment where provide an immunosurveillance for malignant cells by inducing cell death through the surface death receptors. When the death receptors are deficient in the cell surface, a malignant cell could escape from the cytokine-mediated surveillance, which promotes tumor formation and metastasis. As such, the oncogenic activity of H-Ras may be at least partly through its ability in protecting cancer cells from cytokine-mediated apoptosis.

In summary, cancer cells can resist to TRAIL-mediated killing via upregulation of H-Ras. H-Ras effects the subcellular localization of death receptors (DRs), thereby modulating the cellular susceptibility to the DR-targeted therapies. These data provide a rationale for testing the therapeutic potential of combinational drugs that simultaneously block EGFR/Ras-dependent cell growth and activate the death receptor-mediated apoptosis. This distinct function of H-Ras highlights the need of isoform-specific inhibitors in the development of novel cancer therapies.

## MATERIALS AND METHODS

### Cell lines and reagents

The NCI60 panel of human cancer cell lines used for screening TRAIL sensitivity was maintained at the U.S. National Cancer Institute (NCI). RPMI8226 (leukemia), RXF393 and 786-O (renal cancer), and SF539 (CNS) on the NCI60 panel were obtained from NCI through a Material Transfer Agreement. BT549 and T47D (breast cancer) and A549 (non-small cell lung cancer) cell lines were from American Type Culture Collection (ATCC). All cell lines were cultured as per the supplier's recommendations and tested monthly for potential mycoplasma contamination. Recombinant human TRAIL (Cat# 375-TL-010) was from R&D systems (Minneapolis, MN), containing 168 amino acids that correspond to the extracellular domain of human TRAIL (Val114–Gly281), expressed by *E. coli* and purified as a homotrimeric protein. Farnesyl thiosalicylic acid (FTS, Cat# 10010501) and 2-Fluoropalmitic acid (2F-PA, Cat# 90380) were from Cayman (Ann Arbor, MI). Monoclonal antibody specific to human H-Ras (Cat#610002) and Bcl-2 were from BD Biosciences (San Jose, CA). Monoclonal antibodies against human K-Ras (OP24), N-Ras (OP25) or PARP (AM30) were from Calbiochem (Billerica, MA). Antibodies against human caspase 3 (8G10) and caspase 8 (1C12) were from Cell Signalling (Danvers, MA). Antibodies specific to human DR4 (IMG-175), DR5 (IMG-120A), DcR1 (IMG-245-2), DcR2 (IMG-121-1) were from Imgenex (San Diego, CA). Phycoerythrin (PE)-conjugated anti- DR4 (FAB347P), anti-DR5 (FAB6311P), and anti-DcR1 (FAB6302P) were from R & D Systems (Minneapolis, MN). Antibodies to human actin (SC-1616) and Mcl-1 (SC-819) were from Santa Cruz (Dallas, TX). The validated small interference RNA (siRNA) duplexes targeting H-Ras (5′-CCACUAUAGAGGAUUCCUACCGGAA-3′ and 5′-UUCCGGUAGGAAUCCUCUAUAGUGG-3) and K-Ras (5′-CUAUGGUCCUAGUAGGAAA-3′ and 5′-UUUCCUACUAGGACCAUA-3) were purchased from Invitrogen. Transfection of siRNA duplexes were carried out using Neon electroporation transfection system (Invitrogen). Typically, 1-2 μg siRNA duplex was used in 6-well plate format and incubated for 24-48 h. The expression plasmid pcDNA3.1/H-Ras encoding HA-tagged wild-type H-Ras protein was obtained from University of Missouri-Rolla cDNA resource center. Transfections were performed using Lipofectamine LTX (Invitrogen) per manufacturer's instruction.

### TRAIL cytotoxicity

The NCI60 panel anticancer drug screen was carried out at the NCI/NIH Developmental Therapeutics Program (*see* details at http://dtp.nci.nih.gov/branches/btb/ivclsp.html). Briefly, cells were seeded in 96-well plates at plating densities ranging from 5,000 to 40,000 cells per well depending on the doubling time of individual cell lines. After 24-hour incubation, some of the wells were processed to determine a time zero density. To the rest of the plates, rhTRAIL was added at 100 ng/mL and incubated for another 24 hours. The resultant cells were fixed with trichloroacetic acid, stained with sulforhodamine B (SRB), and measured for absorbance at 515 nm. SRB binds to proteins at basic amino acid residues and is used for measuring relative total protein amount and cell viability. Growth inhibition was calculated relative to cells without drug treatment and the time zero control.

### Bioinformatic analysis of NCI60 gene expression data

GeneSpring version 11 software from Agilent Technologies (Santa Clara, CA) was used to analyze the association between the mRNA expression data of each cell line of the NCI60 panel and their respective sensitivity to TRAIL-induced growth inhibition. The genomic mRNA expression data for 58 of 60 cell lines in the NCI60 panel (GSE 5720) were obtained from Shankavaram and colleagues [23]. Average values of replicate spots of each mRNA were background subtracted, and normalized against the median of the control samples. For statistical analysis, the 58 cell lines were first divided into two groups based on their growth inhibition (GI) % values using 50% as a cutoff. The mean expression value of each gene was compared between the two groups using one-way analysis of variance (ANOVA). The genes with a P value < 0.01 in difference between the two groups were picked as candidates for further evaluation. To refine the genes, the 58 cell lines were further divided into 10 groups with a 10% growth inhibition increment on a 0-100% scale. The genes with P value < 0.0001 in difference were considered as signature genes associated with TRAIL resistance.

### Apoptosis Assays

Apoptosis was determined by flow cytometry as previously described [14, 18]. Briefly, cells were grown on 6-well plates to 70-80% confluence and treated with rhTRAIL or anti-DR5 antibody or combination with Ras inhibitors at the indicated concentrations. At the indicated time points, cells were harvested, labeled with FITC-conjugated Annexin V and propidium iodide (PI), and analyzed by FACSCalibur and CellQuest software (Becton Dickinson).

### Cell surface expression of TRAIL receptors

The expression of TRAIL receptors on surface membrane was assessed by flow cytometry using PE-conjugated antibodies (R&D Systems) as previously described [14, 18]. Briefly, cells were grown at 70% - 80% confluence and harvested by incubation with Trypsin (0.25% w/vol)-EDTA (0.53 mM) at 37 °C for 3 min. Trypsinization prevents the formation of cell clumps after harvesting which facilitates the subsequent flow cytometry analysis. The resulting cells were immediately washed twice in PBS. Aliquots (1 X 10^5^cells) were incubated in 25 μL PBS containing 1% goat serum for 15 min at room temperature. Afterwards, cells were incubated with 10 μg/mL anti-DR4-PE or anti-DR5-PE (mouse IgG1-PE and IgG2b-PE as respective control) for 45 min at 4°C in the dark. Duplicate samples were incubated with the respective control IgG-PE (mouse IgG1-PE and IgG2b-PE) under the same conditions. Cells were then washed twice with PBS and resuspended in 0.5 mL PBS for final analysis. All cell lines were processed under the same conditions which ensure a direct comparison of the relative expression levels of DR4 and DR5 on surface membrane.

### Western Blotting

Western blot analyses were done as described [14, 18]. In brief, cells (1 X 10^6^) were lysed in SDS lysis buffer containing 50 mmol/L Tris-HCl (pH 7.0), 2% SDS, and 10% glycerol and incubated for 20 min at 95°C. Protein concentrations were estimated using the BCA protein assay (Pierce, Rockford, IL). Equal amounts of cell lysates (20 μg/lane) were resolved by electrophoresis using a 4% to 12% NuPAGE Bis-Tris gel (Invitrogen) and transferred to PVDF membranes (Millipore) for immunoblot analysis with an appropriate dilution of antibodies (1:1,000-1:2,000). When necessary, the membranes were stripped by Restore Western Blot Stripping Buffer (Pierce) and reprobed with appropriate antibodies. Immunocomplexes were visualized by chemiluminescence using ECL (Santa Cruz).

### Ras activity assay

The relative activity of Ras GTPase was assessed by a commercial Ras activation assay kit (NewEast Biosciences, Malvern, PA). Briefly, cells were grown to 70–80% confluency and subsequently lysed with ice-cold buffer containing 10 mM Tris, 100 mM NaCl, 1% Triton X-100, 0.5 mM ethylenediaminetetraacetic acid, 40 mM β-glycerophosphate, 10 mM MgCl_2_, 1 mM Na_3_VO_4_, 10 μg/ml aprotinin, 10 μg/ml leupeptin and 1 mM phenylmethylsulfonyl fluoride. Cellular debris was removed by centrifugation and total protein concentrations was measured by a colorimetric assay method (Bio-Rad, Hercules, CA). Equal amounts (1.0 mg total protein) of lysates were then incubated at 4°C with an antibody that specifically recognizes the active form of H-Ras (Ras-GTP). After 30 min, protein A agarose beads were added and incubated for an additional 30 min. Next, the beads were collected by centrifugation and were washed three times in the lysis buffer. The beads were boiled in protein loading buffer, and the released proteins were resolved on 12% SDS-PAGE gels and were transferred onto an Immobilon-P membrane (Millipore Corporation, Bedford, MA). The presence of H-Ras-GTP was detected using an anti-H-Ras antibody.

### Statistical analyses

Statistical analyses were performed with SigmaPlot (Systat Software Inc., San Jose, CA). Statistical comparisons were determined by Student's t-test. Statistical significance was defined as *P* < 0.01. Data were presented as mean + SD.

## SUPPLEMENTARY MATERIAL


