# Supplementary material for: H-Ras regulation of TRAIL death receptor mediated apoptosis
Source: Oncotarget. 2014 Jun 11;5(13):5125–37. doi: 10.18632/oncotarget.2091 (PMC4148127; doi:10.18632/oncotarget.2091)

H-Ras regulation of TRAIL death receptor mediated apoptosis

Supplementary Material

Supplement I Western blots of proteins related to caspase activation

Cells were cultured to ~ 80% confluence and whole cell lysateas were analyzed by Western blotting with antibodies specific to the indicated proteins.

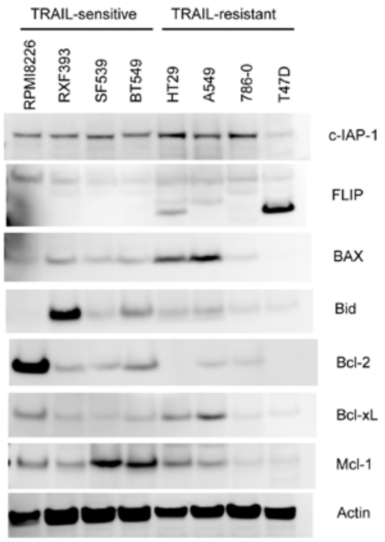

Supplement II Inhibition of pamitolyation sensitized TRAIL-induced cells to TRAIL-induced apoptosis

Cells were pretreated with 2-Fluoropalmitic acid (2F-PA) at 100 microM for 24 h followed by incubation with 10 ng/mL TRAIL for an additional 24 h. The resultant cells were analyzed by immunoblotting and flow cytometry.

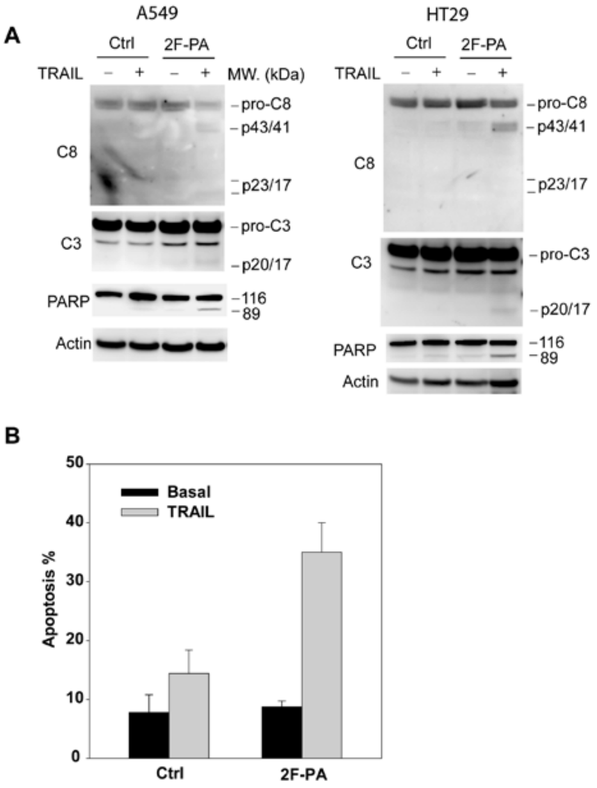

Supplement: Supplementary file 1 [file oncotarget-05-5125-s001.pdf]
